# Alterations of degree centrality and functional connectivity in classic trigeminal neuralgia

**DOI:** 10.3389/fnins.2022.1090462

**Published:** 2023-01-09

**Authors:** Hao Liu, Ruiping Zheng, Yong Zhang, Beibei Zhang, Haiman Hou, Jingliang Cheng, Shaoqiang Han

**Affiliations:** ^1^Department of Magnetic Resonance Imaging, The First Affiliated Hospital of Zhengzhou University, Zhengzhou, China; ^2^Key Laboratory for Functional Magnetic Resonance Imaging and Molecular Imaging of Henan Province, Zhengzhou, China; ^3^Engineering Technology Research Center for Detection and Application of Brain Function of Henan Province, Zhengzhou, China; ^4^Department of Neurology, The First Affiliated Hospital of Zhengzhou University, Zhengzhou, China

**Keywords:** classic trigeminal neuralgia, degree centrality, functional connectivity, resting-state functional MRI, neuropathic pain

## Abstract

**Objectives:**

Recent neuroimaging studies have indicated a wide range of structural and regional functional alterations in patients with classic trigeminal neuralgia (CTN). However, few studies have focused on the intrinsic functional characteristics of network organization in the whole brain. Therefore, the present study aimed to characterize the potential intrinsic dysconnectivity pattern of the whole brain functional networks at the voxel level using the degree centrality (DC) analysis in CTN patients.

**Methods:**

Thirty-four patients with CTN and twenty-nine well-matched healthy controls (HCs) participated in this study. All subjects underwent resting-state functional magnetic resonance imaging (rs-MRI) examination and clinical and neuropsychologic assessments. DC is a graph theory-based measurement that represents the overall functional connectivity (FC) numbers between one voxel and other brain voxels. We first investigated brain regions exhibiting abnormal DC, and further identified their perturbation on FC with other brain regions using a seed-based FC analysis in patients with CTN. In addition, correlation analyses were performed to evaluate the relationship between the abnormal DC value and clinical variables in CTN patients.

**Results:**

Compared with the HCs, the patients with CTN exhibited significantly greater DC values in the right pallidum and right putamen, and lower DC values in the right lingual gyrus, right calcarine sulcus, left paracentral lobule, and left midcingulate cortex. A further seed-based FC analysis revealed that the right lingual gyrus showed decreased FC within the visual network and with other core brain networks, including the sensorimotor network, default mode network, and salience network, relative to HCs. Additionally, the left midcingulate cortex exhibited decreased FC within the middle cingulate cortex and the visual network in CTN patients. Moreover, the DC value in the left midcingulate cortex was negatively correlated with the illness duration.

**Conclusion:**

The present study shows that CTN patients exhibited specific functional connectivity network alterations in the basal ganglia, visual network, and salience network, which may reflect the aberrant neural network communication in pain processing and modulation. These findings may provide novel insight for understanding the mechanisms of pain chronicity in CTN patients.

## 1. Introduction

Classic trigeminal neuralgia (CTN) is a severe neuropathic pain disorder that is limited to the trigeminal territory (Scholz et al., 2019). With the progress of the disease, pain attacks become more frequent and sustained, which severely affects the patient’s physical and mental health, and greatly reduces the quality of life (DeSouza et al., 2016). According to the diagnosis criteria of the International Classification of Headache Disorders (ICHD-3; Headache Classification Committee of the International Headache Society, 2013), the etiology of CTN is largely attributable to the neurovascular compression at the root entry zone. However, we prefer to explore changes in pain processing and information transmission in the brain to help us better understand the process of chronic pain. Identification of brain regions that participate in pain processing, modulation, and chronification in CTN patients will be of great significance to improve the treatment and prognosis.

Recently, accumulating neuroimaging evidence support that CTN is accompanied by altered structure and functional activity of the brain regions engaged in the perception, affective-cognitive, motor function, and modulatory aspects of pain (Xiang et al., 2019; Yan et al., 2019; Zhang et al., 2021). Voxel-based morphometry studies in CTN patients have identified altered gray matter volume and cortical thickness in multiple brain regions, including the thalamus, basal ganglia, primary/secondary somatosensory cortices, and insular cortex (Desouza et al., 2013; Tang et al., 2020). Diffusion tensor imaging studies have revealed widespread white matter fiber abnormalities (Wang et al., 2017; Liu et al., 2018). Meanwhile, abnormal functional activities in multiple cortical and subcortical regions have been identified in CTN patients (Tsai et al., 2018; Chen Y. et al., 2019). This inference is consistent with the notion that the brain is a complex structural and functional brain network, which is believed to constitute the physiological basis for information processing and mental representations (Bullmore and Sporns, 2009). Therefore, these imaging studies support the novel idea that CTN might be a network disorder, and regional abnormalities represent the changes in the network’s nodes. However, previous studies mainly focused on the local structure and functional activity alterations. Few studies aimed to characterize the functional network pattern across the whole brain at the voxel level in CTN patients.

The degree centrality (DC), as a network measurement metric based on graph theory, is capable of assessing the whole brain functional network topological properties (Zuo et al., 2012). The voxel-wise DC represents the number of functional connections between a voxel and the rest of the whole brain voxels. Therefore, it can evaluate the ability of a node to integrate information across functionally segregated brain regions within the network. This method has demonstrated a high level of sensitivity, specificity, and test–retest reliability (Zuo and Xing, 2014). DC has been used to explore the neurobiological mechanism underlying brain network alterations in various neurological diseases (Guo et al., 2016, 2020; Liao et al., 2021), but it has rarely been applied in CTN. Therefore, the voxel-wise DC can be used as an effective approach to investigate the neural alterations in the whole-brain functional networks, which may be in favor of understanding the pathophysiology of CTN.

In the present study, we aimed to characterize the intrinsic dysconnectivity pattern in whole-brain functional networks at the voxel level in CTN patients. First, the voxel-wise DC was performed to identify the voxels that exhibited altered functional connectivity (FC) with other voxels. Next, the seed-based FC approach was applied to evaluate the connectivity abnormality using the regions that showed significant alterations in DC as seeds. In addition, we also investigated the relationships between the altered DC indices and clinical features in CTN patients.

## 2. Materials and methods

### 2.1. Participants

This study was approved by the Ethics Committee of the First Affiliated Hospital of Zhengzhou University. All patients completed formal written consent prior to the MR scanning. This study prospectively recruited thirty-four patients with CTN, and twenty-nine healthy controls (HCs) matched by age and gender. CTN was diagnosed according to the ICHD-3 (Headache Classification Committee of the International Headache Society, 2013) by two experienced neurologists.

Inclusion criteria of CTN patients included: (1) age >18 years; (2) unilateral pain restricted to one or more branches of the trigeminal nerve; (3) paroxysmal, electric shock-like, shooting or stabbing pain, that occurs spontaneously or is activated by normally innocuous mechanical stimuli or orofacial movements; and (4) absence of obvious sensory loss. Exclusion criteria included: (1) other primary headache disorders; (2) surgical history, especially microvascular decompression for CTN; (3) severe somatic or psychiatric disorders; and (4) contraindications to MRI scan. All participants were right-handed. Most of the patients took carbamazepine for pain treatment, a minority used oxcarbazepine or phenytoin. Details about medicine use history can be seen in Table 1.

**TABLE 1:** Demographic and clinical characteristics of the subjects.

Variables	CTN (*n* = 34)	HCs (*n* = 29)	*t*/χ^2^	*P*-value
Age (years)	53.06 ± 10.91	54.21 ± 6.33	-0.520	0.606
Gender (male/female)	16/18	15/14	0.136	0.712
Duration of TN (years)	4.63 ± 3.53	NA		
VAS pain rating	7.97 ± 1.42	NA		
Side affected (L/R), *n*	14/20	NA		
Score of HAMA	8.56 ± 6.01	3.93 ± 2.92	3.98	0.001
Score of HAMD	10.62 ± 6.73	4.6 ± 2.27	4.88	0.001
Mean FD	0.136 ± 0.013	0.057 ± 0.011	-0.979	0.331
Medication	Carbamazepine (28) oxcarbazepine (4) phenytoin (2)	NA		

CTN, classic trigeminal neuralgia; HCs, healthy control subjects; VAS, visual analog scale; HAMA, Hamilton Anxiety Rating Scale; HAMD, Hamilton Depression Rating Scale; NA, not applicable.

### 2.2. Questionnaires and ratings

All patients were instructed to measure their pain intensity using a visual analog scale (VAS) in the last 7 days. The VAS ranges from 0 (no pain) to 10 (worst imaginable pain). The average score was calculated. Depression and anxiety were assessed by the Hamilton Depression Rating Scale (HAMD) and the Hamilton Anxiety Rating Scale (HAMA). All questionnaire evaluation was conducted under the supervision of experimenters.

### 2.3. MRI data acquisition

Imaging data were acquired using a 3.0-T scanner (Discovery 750 System, Milwaukee, WI, USA). All participants laid down on the scanning bed with earplugs and foam padding used to attenuate noise and reduce head motion. The participants were instructed to stay awake and relaxed and to keep their eyes closed without falling asleep during the MRI scan. To rule out the possibility of asymptomatic lesions, a T2-weighted imaging sequence was acquired in all participants. High-resolution three-dimensional structural images were obtained using the following parameters: time of repetition (TR) = 8.15 ms, Time of inversion = 450 ms, time of echo (TE) = 3.17 ms, field of view (FOV) = 256 × 256 mm, slice thickness = 1 mm, matrix = 256 × 256, spatial resolution = 1.00 × 1.00 mm, flip angle = 12.0°. The Resting-state fMRI data were acquired using the single-shot echo planar imaging sequence with the following parameters: TR = 2000 ms, TE = 30 ms, FOV = 220 × 220 mm, flip angle = 90°, in-plane matrix = 64 × 64, spatial resolution = 3.44 × 3.44 × 4 mm, 32 axial slices without slice gap, and a total of 180 volumes for each subject.

### 2.4. MRI data preprocessing

All resting-fMRI data preprocessing was performed using Data Processing Assistant for Resting-State fMRI package (DPARSFA).^1^ The first 10 volumes were discarded to remove initial transient effects and to allow the participant to adjust to the scanner noise. Then, the remaining functional images were corrected with slice timing for the acquisition delay between slices and realignment. The mean frame-wise displacement (FD) was calculated for each subject (Power et al., 2012; Han et al., 2020). Subjects were excluded if the translational and rotational displacement exceeded 3.0 mm or 3.0° between successive volumes. The functional images were spatially normalized to the standard EPI template and resampled to 3 mm × 3 mm × 3 mm. Subsequently, the normalized functional images were further smoothed using an isotropic Gaussian filter (6-mm FWHM) and detrended to reduce low-frequency drift. Next, several nuisance covariates, including white matter signals, cerebrospinal fluid signals, and Friston-24 head motion parameters (Satterthwaite et al., 2012), were removed by linear regression. Then temporal band-pass filter (0.01–0.08 Hz) was performed. Scrubbing with cubic spline interpolation was used to mitigate the influence of head motion and ensure the contiguous time points.

### 2.5. Voxel-wise DC analysis

The voxel-wise DC value calculations were conducted using the DPABI software based on preprocessed data, as has been well described in previous studies (Guo et al., 2016; Li et al., 2016). Pearson’s correlation coefficients (r) were computed between the time course of a given voxel and all other whole-brain voxels within the whole-brain gray matter mask. The whole-brain functional network was constructed with the correlation threshold being set at *r* ≥ 0.25 (Buckner et al., 2009). To increase the stability and repeatability, another two different correlation thresholds (*r* ≥ 0.2 and 0.3) were analyzed. Then, the generated DC value mapping was normalized with Fisher’s r-to-z transformation to construct the Z-score DC value map, which was used for further analysis.

### 2.6. Functional connectivity analysis

Following the DC analyses, seed-based resting-state FC was conducted using the temporal correlation approach. The brain regions that exhibited significantly altered DC in CTN patients were chosen as seeds. The seed regions were generated by drawing a 6-mm radius sphere centered on the peak voxels of regions with a significant difference in DC between CTN patients and HCs. The time series for seeds were obtained by averaging the time series of all voxels within the seed region. Pearson’s correlation analyses were calculated between the averaged time series from each seed and the rest of the brain. The correlation map was obtained for each subject. Then, the correlation map was transformed to Fisher *z*-values to get a z-FC map for further statistical analysis.

### 2.7. Statistical analysis

SPSS software version 23.0 (IBM Corporation, Armonk, NY, USA) was applied for the demographic and clinical data analyses. A two-sample *t*-test was used to test for the continuous variables (age, HAMA and HAMD scores). The Chi-square test was used to test the gender proportion between CTN patients and HCs subjects. The significant level was set as *p* < 0.05. Then, a two-sample *t*-test was performed to evaluate differences in DC between the CTN patients and HCs groups, with age, gender, and mean FD as covariates using SPM12. Multiple comparisons were corrected by the false discovery rate (FDR) approach (*p* < 0.05).

For the seed-based FC map, a two-sample *t*-test was applied to investigate the differences between the two groups, with age, gender, and mean FD as covariates using SPM12. Then the analyses were corrected for multiple comparisons using the FDR (*p* < 0.05). The threshold for the cluster size is set to 100 voxels.

### 2.8. Correlation analysis with clinical variables

We performed a two-tailed partial correlation analysis to further assess the relationship between the DC values of altered brain regions and clinical variables (VAS scores, disease duration, HAMD, and HAMA scores) in the CTN group, controlling for age, gender, and mean FD. The statistical significance level of *p* < 0.05 was set for all correlation analyses.

## 3. Results

### 3.1. Demographic and clinical features

There were no significant differences in age and gender (*p* > 0.05) between the CTN and HC groups, while CTN patients had significantly higher HAMA and HAMD scores (*p* < 0.05) compared with HCs.

### 3.2. Alterations of DC between groups

**FIGURE 1:**
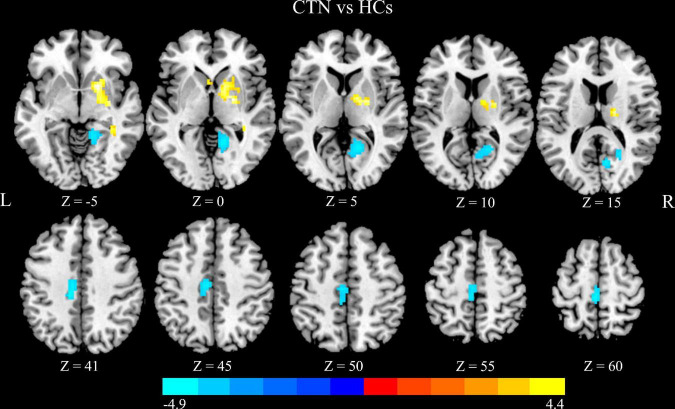
Altered DC in CTN patients. Compared with HCs, the patients with CTN showed greater DC values in the right pallidum and right putamen, and lower DC values in the right lingual gyrus, right calcarine sulcus, left paracentral lobule, and left midcingulate cortex (*p* < 0.05, FDR corrected). The warm colors indicate a higher DC value whereas the cooler colors indicate a lower DC value. The color bar indicates *T*-value. DC, degree centrality; CTN, classic trigeminal neuralgia; HCs, healthy control subjects; FDR, false discovery rate.

Compared with HCs, CTN patients showed significantly greater DC values in the right pallidum and right putamen, and lower DC values in the right lingual gyrus (LG), right calcarine sulcus, left paracentral lobule and left midcingulate cortex (MCC) (*p* < 0.05, FDR corrected; Figure 1 and Table 2). The intergroup differences were also similar at different correlation thresholds (0.2, 0.25, and 0.3) (Supplementary Figure 1).

**TABLE 2:** Brain regions with significant differences in DC values between CTN patients and HCs.

Anatomical regions	MNI coordinates			Voxels in cluster	Volumes (mm^3^)	Peak *t* value
	**x**	**y**	**z**			
Pallidus _R	21	−3	−3	54	1458	4.34
Putamen _R	21	9	−6	77	2079	4.36
Paracentral lobule _L	0	−33	60	59	1593	-3.25
Cingulum _Mid _L	−12	−21	39	98	2646	-3.27
Calcarine _R	24	−57	9	83	2241	-3.26
Lingual _R	21	−45	−6	93	2511	-3.36

DC, degree centrality; CTN, classic trigeminal neuralgia; HCs, healthy control subjects; MNI, Montreal Neurological Institute; L, left; R, right; Mid, middle.

### 3.3. Alterations of seed-based resting-state FC between groups

**FIGURE 2:**
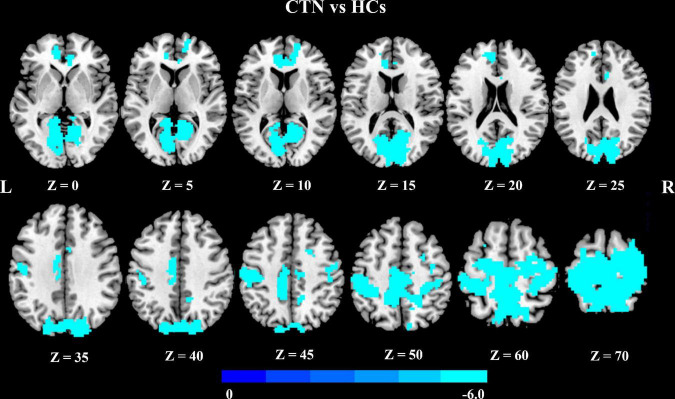
Altered right lingual gyrus FC in CTN patients. Compared with HCs, the CTN patients exhibited significantly decreased FC between the right lingual gyrus and sensorimotor network, default mode network, and salient network. The significance threshold was set at *p* < 0.05 with FDR correction (cluster extent threshold >100 voxels). The cooler colors indicate a lower FC value. The color indicates *T*-value. FC, functional connectivity; CTN, classic trigeminal neuralgia; HCs, healthy control subjects; FDR, false discovery rate.

We further explored seed-based FC associated with six regions of interest (right pallidum, right putamen, right LG, right calcarine sulcus, left paracentral lobule, and left MCC). Notably, compared with HCs, CTN patients showed significantly decreased resting-state FC between the right LG and multiple brain areas, including bilateral precentral gyrus, bilateral postcentral gyrus, bilateral supplementary motor area, bilateral paracentral lobule, bilateral precuneus, bilateral calcarine gyrus, bilateral LG, bilateral cuneus gyrus, bilateral superior occipital gyrus, right superior parietal gyrus, right superior frontal gyrus, anterior cingulate cortex, and MCC (*p* < 0.05, FDR corrected; Figure 2 and Table 3).

**TABLE 3:** Brain regions with significant differences in FC of right lingual gyrus between the CTN patients and HCs.

Seed region	Target regions	Side	MNI coordinates			Voxels in cluster	Volumes (mm^3^)	Peak *t* value
			***x***	***y***	***z***			
LG_R	Cluster 1							
	Paracentral lobule	L/R	−6	−33	51	427	11529	−6.0234
	Postcentral gyrus	L/R				825	22275	
	Precentral gyrus	L/R				394	10638	
	Supplementary motor area	L/R				266	7182	
	Precuneus	L/R				683	18411	
	Calcarine gyrus	L/R				579	15633	
	Lingual gyrus	L/R				497	13419	
	Cuneus gyrus	L/R				485	13095	
	Superior occipital gyrus	L/R				339	9153	
	Superior parietal gyrus	R				141	3807	
	Superior frontal gyrus	R				137	3699	
	Middle cingulate cortex	L				181	4887	
	Cluster 2							
	Anterior cingulate cortex	L	−12	45	0	129	3483	−4.9272

FC, functional connectivity; CTN, classic trigeminal neuralgia; HCs, healthy control subjects; MNI, Montreal Neurological Institute; L, left; R, right.

**FIGURE 3:**
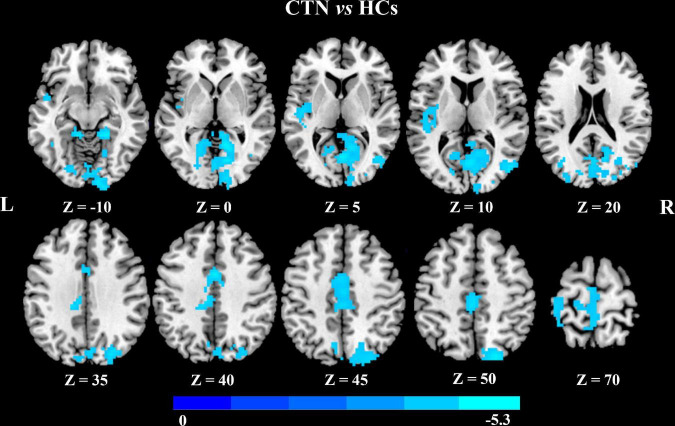
Altered left midcingulate cortex FC in CTN patients. The CTN patients showed significantly decreased FC between the left midcingulate cortex and visual network, left paracentral lobule compared with HCs (*p* < 0.05, FDR corrected, cluster >100 voxels). FC, functional connectivity; CTN, classic trigeminal neuralgia; HCs, healthy control subjects; FDR, false discovery rate.

In addition, seed-based FC analysis showed that CTN patients exhibited significantly decreased FC between left MCC and bilateral calcarine gyrus, bilateral LG bilateral cuneus gyrus, right middle occipital gyrus, right superior occipital gyrus, left MCC, and left paracentral lobule, compared with HCs (*p* < 0.05, FDR corrected; Figure 3 and Table 4).

**TABLE 4:** Brain regions showing FC differences based on left MCC between the CTN patients and HCs.

Seed region	Target regions	Side	Peak MNI coordinates			Voxels in cluster	Volume (mm^3^)	Peak *t* value
			***x***	***y***	***z***			
MCC	Cluster 1							
	Calcarine gyrus	L/R	18	−57	−3	490	13230	−5.3392
	Lingual gyrus	L/R				382	10314	
	Cuneus gyrus	L/R				254	6858	
	Middle occipital gyrus	R				107	2889	
	Superior occipital gyrus	R				130	3510	
	Cluster 2							
	Middle cingulate cortex	L	0	3	39	147	3969	−5.0442
	Paracentral lobule	L				132	3564	

FC, functional connectivity; MCC, midcingulate cortex; CTN, classic trigeminal neuralgia; HCs, healthy control subjects; MNI, Montreal Neurological Institute; L, left; R, right.

### 3.4. Correlation analysis between clinical variables and DC

**FIGURE 4:**
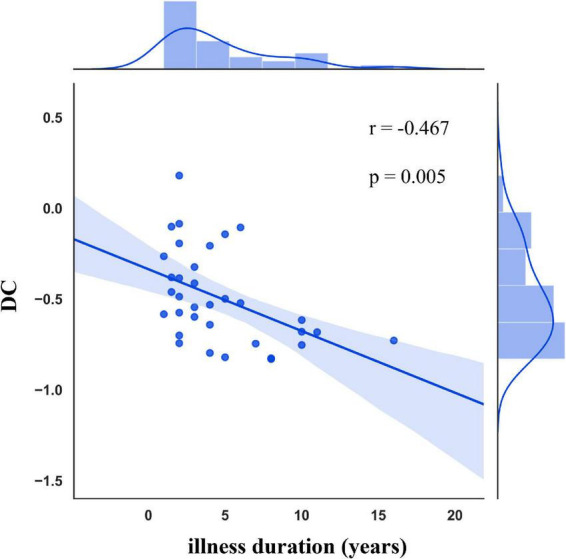
Correlation between DC alterations and clinical variables in CTN patients. DC in the left MCC was negatively associated with the illness duration. DC, degree centrality; CTN, classic trigeminal neuralgia; MCC, midcingulate cortex.

In patients with CTN, there was a negative correlation between DC values in left MCC and illness duration (r = −0.467, *p* = 0.005; Figure 4). However, there was no significant association between DC values and other clinical parameters.

## 4. Discussion

Our study found that CTN patients had greater DC values in the right pallidum and right striatum, and lower DC values in the left paracentral lobule, left MCC, right LG, and right calcarine sulcus, compared with HCs. Furthermore, compared with HCs, CTN patients exhibited disrupted FC within the visual cortex and with other brain regions, mainly including the precentral gyrus, postcentral gyrus, supplementary motor area, precuneus, anterior cingulate cortex, and MCC. CTN patients also showed decreased FC between the left MCC and bilateral visual cortex, left MCC, and left paracentral lobule. These findings provide novel insight into the large-scale functional reorganization that occurs in CTN patients. Moreover, DC values in left MCC were significantly negatively correlated with illness duration, which indicated that the dysfunction of MCC may contribute to pain chronification in CTN patients.

Degree centrality represents the overall connectivity numbers between one voxel and other brain voxels (Buckner et al., 2009; van den Heuvel and Sporns, 2013). In this study, CTN patients exhibited lower DC values in the right LG and calcarine sulcus, which are core hubs of visual network (VIN). Accumulating evidence has indicated that VIN is not only involved in visual processing and multisensory integration (Tong, 2003) but also participates in different high-order functions and directly relates to perception and behavior (Murray et al., 2016; Huang and Zhu, 2017). Consistent with our study, altered regional activities in the occipital gyrus have been reported in CTN patients (Yuan et al., 2018; Chen Z. et al., 2019). However, Zhu et al. (2020) have found higher DC values in the right LG, which differs from our study. The most likely explanation for this difference might be the heterogeneity of patients, as the patients in our study had longer disease duration and more severe pain.

In addition, further seed-based FC analysis disclosed that CTN patients had decreased FC between right LG and several brain regions, which were mainly located in the sensorimotor network (SMN), VIN, default mode network (DMN), and salient network (SN) (Beckmann et al., 2005; Menon and Uddin, 2010; Raichle, 2015). Many studies have demonstrated that SMN plays a crucial role in the pain process and modulation in CTN patients (Tsai et al., 2018; Henssen et al., 2019). The attenuated FC between LG and SMN might be an adaptive response to persistent nociceptive input or to an inhibition of jaw movement to avoid eliciting pain (Svensson and Graven-Nielsen, 2001). Another possible, more likely, explanation is that there is a disruption in FC simply because the CTN patients are in pain. Since the painful incoming sensations from the trigeminal nerve of the face would activate a large area of the SMN and the LG is displaying a different pattern of activation, therefore there is less FC compared to HCs because there are two distinct patterns of activity in each part of the cortex. Moreover, we found decreased FC between the right LG and the bilateral precuneus. Precuneus is a critical node of the DMN that is involved in visuospatial processing, episodic memory, self-reflection, and consciousness (Hebscher et al., 2019). Furthermore, in the theory of the dynamic pain connectome, the DMN and SN are crucial components of the pain connectome and key contributors to the ongoing dynamics of pain (Kucyi and Davis, 2015). Therefore, given that CTN is characterized by long-term paroxysmal attacks, the disrupted visual-related FC patterns probably reflected an interruption of switching between internal and external stimuli and affected the pain-related perception, regulation, and emotional, cognitive, and sensorimotor aspects of pain (Zou et al., 2021).

Our study revealed that CTN patients had lower DC values in left MCC compared with HCs. It has been reported that MCC was associated with attentional processing and cognitive aspects of pain (Vogt, 2005; Nevian, 2017). Meanwhile, MCC is an important node of SN, which integrates information about the significance of an impending stimulation into perceptual decision-making in the context of pain (Wiech et al., 2010). Changes in the functional activity (Glass et al., 2011; Fallon et al., 2016) and structural integrity of MCC (Erpelding et al., 2016; Wang et al., 2017) have been reported in CTN patients. Furthermore, DC values in left MCC were significantly negatively correlated with illness duration in our study, suggesting that a long history of pain might contribute to brain dysfunction, which indicates that MCC may play a vital role in the pathogenesis of pain chronification and may be responsible for the transition from acute pain to chronic pain in CTN patients.

Additionally, a further seed-based FC analysis demonstrated that CTN patients showed significantly less FC between MCC and several brain regions, which are mainly located in VIN. SN and VIN are components of the sensory system, and their main function is to participate in the information processing of external stimuli (Chen et al., 2021). The less FC between MCC and LG may reduce the ability to process pain-related perception information. Therefore, these findings suggested the FC between MCC and LG may play a crucial role in the pain processing of CTN patients.

In addition to lower DC in multiple brain regions, we also found greater DC in the right pallidum and right putamen, compared with HCs. Putamen and pallidum are the main nodes of the cortex-basal ganglia-cortex loops, which are probably involved in the sensory, motor, emotional, memory, and cognitive aspects of pain (Borsook et al., 2010; Starr et al., 2011). Thereby, the greater DC in basal ganglia revealed in our study may be an adaptive response to long periods of pain attacks, which may be involved in CTN patients limiting their orofacial movements to avoid triggering pain (Bennetto et al., 2007). Consistent with our findings, previous studies have reported altered gray matter volume and spontaneous activities in basal ganglia in CTN patients (Yuan et al., 2018; Tang et al., 2020). Therefore, it is possible that long-lasting pain stimulation in CTN patients results in more demands for pain modulation, which is manifested as relatively higher DC values in these areas. We speculate that the increased functional activity observed in our study probably was an adaptive and compensatory response of the brain to meet the increased demand for pain processing during the recurring attacks of pain in CTN patients.

## 5. Limitation

We recognize some limitations in this study. First, the selection of the threshold for computing DC (*r* ≥ 0.25) in this study is subjective, although the threshold is consistent with previous studies (Buckner et al., 2009; Wang et al., 2018). However, Buckner et al. (2009) found that the selection of different thresholds for the calculation of DC would only have a slight impact on the main findings. Second, this study is a cross-sectional study and did not investigate the dynamic brain functional changes in CTN patients. Therefore, it is not possible to draw causal conclusions between these findings and CTN pain. Third, it is still impossible to rule out the potential impact of anti-epileptic agents on the results. The mechanism of action of carbamazepine seems to be mainly related to the blockade of sodium channels in neuronal membranes during high-frequency stimulation, thereby reducing the propagation of the electrical signal and limiting the spread of ectopic activity. Therefore, we speculate that anti-epileptic agents may have an impact on regional brain activity and cause abnormal FC between various brain regions. Fourth, due to the relatively small sample size, the functional findings presented here were exploratory and preliminary. Large-sample, multi-center, and longitudinal studies should be conducted in the future.

## 6. Conclusion

Our study showed that CTN patients exhibited lower DC values in the right LG, right calcarine sulcus, left paracentral lobule, and left MCC, and higher DC values in the right pallidum and right putamen compared with HCs. Moreover, lower DC in left MCC was associated with illness duration in CTN. Some of those altered DC regions showed abnormal FC with other brain regions that are associated with pain processing. Taken together, these findings suggest that CTN patients are likely characterized by specific whole-brain functional connection alterations, which may underpin the refractory pain in CTN and provide an alternative target for clinical treatments.

## Data availability statement

The raw data supporting the conclusions of this article will be made available by the authors, without undue reservation.

## Ethics statement

The studies involving human participants were reviewed and approved by the Ethics Committee of The First Affiliated Hospital of Zhengzhou University. The patients/participants provided their written informed consent to participate in this study.

## Author contributions

HL, JC, and SH: conception and study design. HL, RZ, and YZ: data collection or acquisition. HL and BZ: statistical analysis. HL, HH, YZ, and SH: interpretation of results. HL, RZ, JC, BZ, and SH: drafting the manuscript work or revising it critically for important intellectual content. All authors approved the final version to be published and agreement to be accountable for the integrity and accuracy of all aspects of the work.

## Footnotes

1
http://www.restfmri.net
