# Supplementary figures and images for: Alterations of degree centrality and functional connectivity in classic trigeminal neuralgia
Source: Front Neurosci. 2023 Jan 9;16:1090462. doi: 10.3389/fnins.2022.1090462 (PMC9870176; doi:10.3389/fnins.2022.1090462)

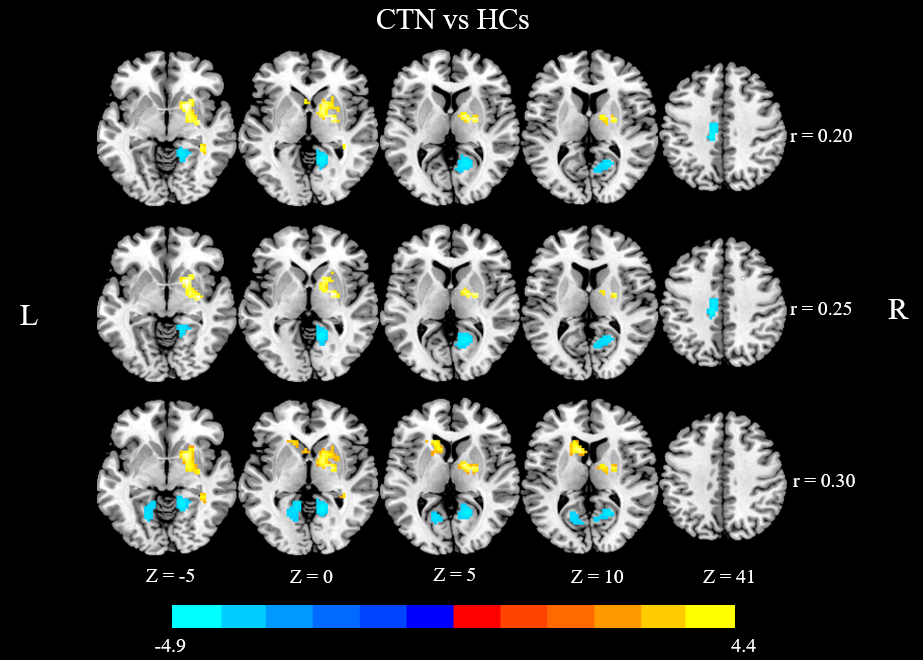

Supplement: Supplementary file 1 [file Image_1.TIF]
